# Choosing a cellular model to study SARS-CoV-2

**DOI:** 10.3389/fcimb.2022.1003608

**Published:** 2022-10-21

**Authors:** Gabriel Augusto Pires De Souza, Marion Le Bideau, Céline Boschi, Nathalie Wurtz, Philippe Colson, Sarah Aherfi, Christian Devaux, Bernard La Scola

**Affiliations:** ^1^ Microbes, Evolution, Phylogeny and Infection (MEPHI), UM63, Institut de Recherche pour le Développement (IRD), Assistance Publique - Hôpitaux de Marseille (AP-HM), Aix-Marseille Université, Marseille, France; ^2^ Institut Hospitalo-Universitaire Méditerranée Infection, Marseille, France; ^3^ Department of Biological Sciences (INSB), Centre National de la Recherche Scientifique, Marseille, France

**Keywords:** SARS-CoV-2, COVID-19, viral culture, *in vitro* approaches, susceptible cells, cell lines, organoids, cell model

## Abstract

As new pathogens emerge, new challenges must be faced. This is no different in infectious disease research, where identifying the best tools available in laboratories to conduct an investigation can, at least initially, be particularly complicated. However, in the context of an emerging virus, such as SARS-CoV-2, which was recently detected in China and has become a global threat to healthcare systems, developing models of infection and pathogenesis is urgently required. Cell-based approaches are crucial to understanding coronavirus infection biology, growth kinetics, and tropism. Usually, laboratory cell lines are the first line in experimental models to study viral pathogenicity and perform assays aimed at screening antiviral compounds which are efficient at blocking the replication of emerging viruses, saving time and resources, reducing the use of experimental animals. However, determining the ideal cell type can be challenging, especially when several researchers have to adapt their studies to specific requirements. This review strives to guide scientists who are venturing into studying SARS-CoV-2 and help them choose the right cellular models. It revisits basic concepts of virology and presents the currently available *in vitro* models, their advantages and disadvantages, and the known consequences of each choice.

## Introduction

In order to study obligate intracellular parasites such as viruses, it is necessary to have the capacity to maintain the host upon which they reproduce in the laboratory. When virology was just in its infancy, at a time when viruses were still understood as filterable infectious agents, the study of viruses was limited to plant viruses and, later, to bacterial viruses (bacteriophages), since cultivating their hosts was relatively easy (Simon, 1912; Bos, 1981; Van Helvoort, 1994; Clokie et al., 2011).

This was a particular limitation for animal viruses, however, since initially experimental animals were the only available means of isolating and multiplying viruses (e.g. the rabies virus was multiplied in rabbits) (Gorman, 1991; Faisst, 1999). One alternative was to inoculate virus samples into the cavities (such as the allantois) of embryonated eggs, where there are tissues which are susceptible to infection by certain viruses (such as measles, influenza, polio, and herpes viruses) (Faisst, 1999).

It was only in the 1940s, with the improvement of cell culture techniques, that the study of animal viruses made significant advances (Faisst, 1999; Leland and Ginocchio, 2007). In 1953, HeLa cells were found to be an effective tool for growing large quantities of poliovirus (Scherer et al., 1953), and this knowledge laid important groundwork for the later development of the polio vaccine (Weller et al., 1949; Sabin and Boulger, 1973). Later, the growth of T lymphocytes from normal human bone marrow and the discovery of interleukin 2 made it possible to characterize the first human retrovirus in the early 1980s (Morgan et al., 1976; Mier and Gallo, 1980; Poiesz et al., 1980). It is now easy to manipulate and clean flasks, grow thousands of cells (in the form of immortalized human and animal cell lines) which are susceptible to virus isolation, generate viruses in high titers, and, in the case of antibiotics, control contamination (Leland and Ginocchio, 2007).

Coronaviruses (CoV) were identified by electron microscopy in the mid-1960s precisely because of the difficulty of multiplying the infectious agent, which was hitherto unknown, in routine cell culture at that time, even though it multiplied *in vitro* in organ cultures. The researchers Tyrrell and Bynoe decided to try to visualise the particles through electron microscopy, a piece of equipment handled by June Almeida (Tyrrell et al., 1965; Almeida and Tyrrell, 1967). As a result, they identified the characteristic particle of coronaviruses with the prominent spikes forming a kind of crown, hence the name of the virus.

Coronaviruses are enveloped viruses with a positive single-stranded RNA genome that infect various animal hosts, including humans. The family *Coronaviridae* comprises subfamily *Orthocoronavirinae* that is divided into four genera: *Alphacoronavirus*, *Betacoronavirus*, *Gammacoronavirus*, and *Deltacoronavirus* (Cui et al., 2019; Murgolo et al., 2021; Zhou et al., 2021c) However, only seven coronaviruses of the *Alpha* and *Beta* genera are known to infect humans (Chu et al., 2020), triggering pathologies that range from typical symptoms of the common cold to life-threatening respiratory illnesses in the lower respiratory tract (Murgolo et al., 2021).

Two of these coronaviruses are considered as being historically relevant, due to the outbreaks they caused in the past, of namely Severe Acute Respiratory Syndrome (SARS) and Middle East Respiratory Syndrome (MERS). SARS was first reported in Asia in February 2003, though cases were subsequently tracked back to November 2002. SARS quickly spread to 26 countries until the epidemic subsided after about four months, with no new cases being no longer detected since 2004. (Li et al., 2005; Graham and Baric, 2010). More than 8,000 people were infected with SARS-CoV, and 774 died (a fatality rate of about 10%) (Ooi and Phua, 2009). MERS-CoV was first reported in Saudi Arabia in September 2012 and has since spread to 27 countries (Ramadan and Shaib, 2019; Al-Tawfiq et al., 2021). The fatality rate of MERS-CoV was much higher (estimated at about 38%), with more than 400 deaths mainly in the Middle East.

In December 2019, a new betacoronavirus named Severe Acute Respiratory Syndrome Coronavirus 2 (SARS-CoV-2) (Gorbalenya et al., 2020; Hui et al., 2020) was detected in patients presenting with viral pneumonia in Wuhan, China (Zhu et al., 2020). SARS-CoV-2 was notable for its rapid spread and quickly became a threat to global public health, and was recognised as potentially leading to the risk of global healthcare system collapse (WHO, 2021). However, unlike previous SARS-CoV and MERS-CoV outbreaks, the new coronavirus outbreak expanded tremendously worldwide. As a result, in March 2020, the World Health Organization (WHO) declared the new coronavirus disease, Coronavirus Disease 2019 (COVID-19), as a pandemic (Murgolo et al., 2021).

After SARS-CoV-2 broke through the borders of Asia and became a global threat, coronaviruses started attracting dramatically increased attention of the scientific community and governments alike. Although much could be inferred from what had been discovered for SARS-CoV and MERS-CoV (Boni et al., 2020; Zhou et al., 2021b), significant doubts remained about the particularities of SARS-CoV-2, including the most suitable cellular models for studying the new coronavirus.

Developing models of infection and pathogenesis was considered an urgent requirement by the scientific community. In order to combat SARS-CoV-2, cell-based approaches are crucial for understanding coronavirus infection biology, growth kinetics, and tropism (Leist et al., 2020). The commonly used laboratory cell lines were the first line of experimental models used to study the viral pathogenicity and to perform assays aimed at screening antiviral compound which were efficient for blocking the replication of emerging viruses (Saccon et al., 2021).

For example, selecting optimal cell line(s) for compound evaluation and screening is imperative for understanding the antiviral mechanisms of action beyond the inhibition of viral non-structural proteins (Murgolo et al., 2021). While assays to determine the neutralizing antibodies titers are performed in virus-specific permissive cells and understood as strong correlate of vaccine efficacies in humans (Earle et al., 2021; Padmanabhan et al., 2022).

However, determining the ideal cell type can be challenging when it comes to studying a new virus, especially in scenarios where several researchers are adapting their studies to meet a specific need. This review aims to guide scientists who are venturing into the study of SARS-CoV-2 and to help them choose cellular models. It revisits basic concepts of virology and presents the currently available *in vitro* models, their advantages and disadvantages, and the known consequences of each choice.

## General concepts involved in choosing cells to study viruses

One of the characteristics of most viruses is that they are host-specific and have a specific tropism. In other words, viruses only can attach to and infect certain cells of certain organisms (known as “susceptible hosts”) which express the appropriate viral receptor(s). This is also why research is conducted into the cell line that will best support the replication of the virus in question and facilitate observation of the phenomena being investigated by the researcher (Lednicky and Wyatt, 2012). Choosing a cellular model to study viruses requires an understanding of certain basic concepts of virus-host interaction. These concepts often become essential to identify a suitable cell model, although many can be established empirically. These are general requirements when it comes to choosing a cell model to study any virus and should also guide the choice of cells for studying SARS-CoV-2.

### Host and cellular tropisms

Understanding that viruses have a specific host range, being species-specific, and a specific cellular tropism is the first step towards finding a cell to study. The most rational way is to look for cells from the same host species from which the virus has been isolated or from a phylogenetically close species. In the case of human viruses, in addition to the wide variety of isolated cells derived from this species, many viruses are produced in non-human primate cells (Kiesslich and Kamen, 2020). Obviously, cells isolated from other animals allow the replication of human viruses (Tuffereau and Flamand, 1983; Lee et al., 2020a). The capability of a virus to infect a distinct group of cells in the host is defined as tropism, which is often associated with a variety of cellular devices available on the surface of the host cell (Chappell and Dermody, 2015).

For example, the oncolytic myxoma virus (MYXV), a member of the *Leporipoxviru*s genus, typically infects rabbits but not humans (McFadden et al., 2009; Rahman and McFadden, 2020). This is understood as a host tropism. As an example of tissue tropism, the influenza virus typically infects lung tissues, while HIV presents cellular tropism for CD4+ T lymphocytes (Weiss, 2002; McFadden et al., 2009; Reperant et al., 2012). The main cell models for studying the hepatitis B and D viruses are carried out in cell lines initially derived from liver tissue or hepatoma cell lines (Hu et al., 2019; Heuschkel et al., 2021). When it comes to antiviral assays for HIV inhibition studies, primary *in vitro* cell culture, particularly monocytes or cells derived therefrom, are usually used as relevant and robust infection models (Wong et al., 2021). Considering viral tropism is essential, therefore, for the development of cellular models for studying viruses.

Although the main manifestations of COVID-19 are observed in the respiratory tract, current evidence points to a multiple organ and cell tropism of SARS-CoV-2 infection. By detecting SARS-CoV-2 antigens in post-mortem samples, SARS-CoV-2 has been found to infect the respiratory system (i.e., the lungs and trachea) but also the kidneys, small intestines, pancreas, blood vessels, and other tissues (Liu et al., 2021). In addition, it has been suggested that SARS-CoV-2 also targets even the sweat glands and vascular endothelial cells in the skin (Liu et al., 2020). The consequences of this broad spectrum of viral tropisms may contribute to multi-organ damage, which is a concern in the pathophysiology of COVID-19.

With the spread of SARS-CoV-2 and the emeregence of new variants, there are strong indications that there has been a shift in tropism in these variants, such as Omicron (B.1.1.529) (Meng et al., 2022). This, associated with the viral tropism of SARS-CoV-2 for various cell types, reflects a differential expression of the key host factors involved in viral attachment and entry (Murgolo et al., 2021). A study using the SARS-CoV-2 Wuhan 1 strain (B lineage), which estimated the relative usage of entry pathways in different cell lines, demonstrated that each cell lineage has a relative percentage of entry preferential pathway mediated by host proteases to be used by the virus (Padmanabhan et al., 2020). While another study suggests that the Omicron variant (B.1.1.529) shows increased Cathepsin B/L mediated entry compared to other strains (Padmanabhan & Dixit, 2022 - Preprint). Unravelling the cellular factors that determine viral tropism is, therefore, an important step for predicting viral pathogenesis and for choosing cellular models to study the virus.

### Cellular receptors and viral entry

The adhesion step must be well orchestrated to overcome cellular barriers. Once it reaches the intracellular environment, the virus can establish the infection and take possession of the cellular machinery. Therefore, virus-receptor interaction is the key to cell invasion (Dimitrov, 2004; Maginnis, 2018; de Souza et al., 2021). For many viruses, the availability of virus receptors on the surface of a host cell determines the tropism (Chappell and Dermody, 2015), and studies on viral receptors have established certain generalities: (1) some viruses (such as lentiviruses) use not only a receptor, but also a coreceptor, and both determine tropism (for example, the human immunodeficiency virus binds to CD4 and uses CCR5, CXCR4 or both as co-receptors); (2) many viruses can use more than one molecule as a receptor (e.g. HIV can enter cells through the DC-sign); (3) viruses are constantly adapting and a single virus can change its receptor usage after successive passages in cell culture or animal models; and (4) the expression pattern of receptors *in vitro* and *in vivo* can be different, which can mislead researchers (Dimitrov, 2004; Neal, 2014; Bhella, 2015).

After the adhesion of the viral particle to the cell receptor, the virus begins the process of entering the cell which will potentially be infected. This process of viral entry can occur in different ways. For animal viruses, this is commonly through endocytosis pathways, especially clathrin-mediated pathways. This is an effective process that transports incoming viruses together with their receptor into endosomes (Marsh and Helenius, 2006). Indeed, other pathways, such as the micropinocytosis pathway (an action-driven process) (Canton, 2018), the clathrin-independent pathway, the caveolar pathway, and the cholesterol-dependent endocytic pathway which is devoid of clathrin and caveolin, have also been identified as an entry mechanism for the viruses (Dimitrov, 2004; Marsh and Helenius, 2006; Sobhy, 2017). In addition, viruses can fuse their membrane to the host membrane, as has been observed for HIV, the measles virus and some poxviruses (Buchholz et al., 1996; Sobhy, 2017).

**Figure 1:**
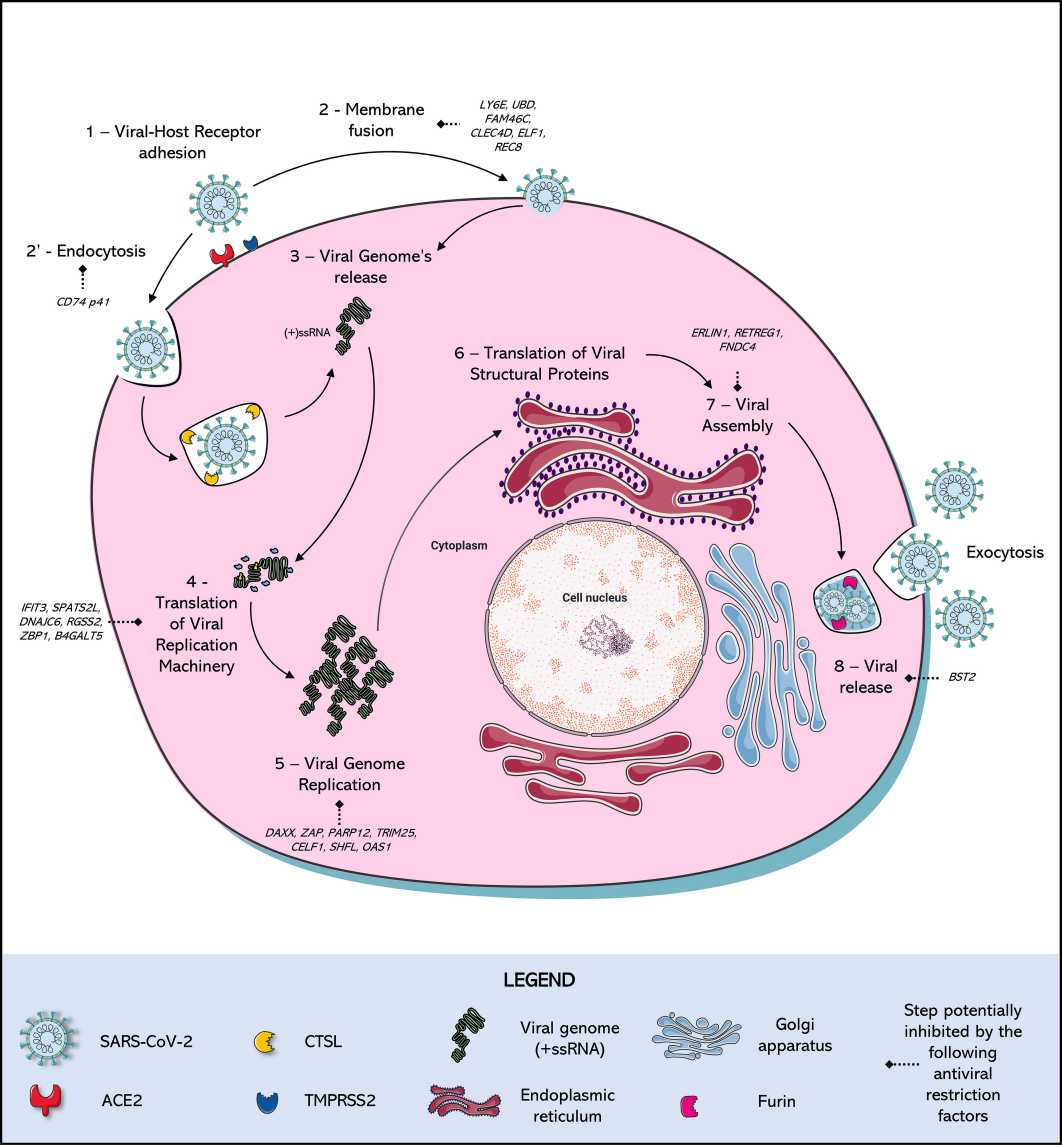
SARS-Cov-2 Replication Cycle and antiviral restriction factors in each step of viral replication. (1) Adhesion: SARS-CoV-2 Spike (S) protein binds to a cellular receptor, which is mostly Angiotensin-Converting Enzyme 2 (ACE2), although alternative receptors are described (Ex: ASGR1, KREMEN1). (2) Entry: When there is expression of transmembrane protease serine 2 (TMPRSS2), this protease cleaves the viral Spike protein mediating entry by fusion of the viral membrane to the host cell membrane. In parallel, in the absence of expression of this protease, entry occurs by (2’) endocytosis mediated by the receptor, triggering the formation of endo-lysosomes in which Cathepsin L (CTSL) will be responsible for the cleavage of the Spike protein. A new conformational arrangement is induced by this cleavage, triggering (3) the viral genome (+ssRNA) release (via uncoating) into the cell cytoplasm. After the viral RNA is delivered into the host cell, the (4) translation of the viral replication machinery begins: the coronavirus genomic RNA encodes nonstructural proteins (NSPs) that have a critical role in (5) Viral Genome replication: process in which the virus induces the synthesis its RNA, mediated by NSPs. (6) Translation of Viral Structural Protein: the structural proteins S, Envelope (E), and Membrane (M) are translated by ribosomes that are bound to the endoplasmic reticulum (ER). The nucleocapsid proteins (N) remain in the cytoplasm and are assembled from genomic RNA. They fuse with the precursor virion, which is then transported from the ER through the Golgi Apparatus. The Spike cleavage at the S1/S2 furin site probably takes place when virions are released through the Golgi apparatus, responsible for the (7) Viral release: transporting virions to the cell surface *via* small vesicles, finally released by (8) Exocytosis: the viral progeny is released by exocytosis to the extracellular medium, ready to find and infect new cells. Many of these steps are antagonized by intact cell defense mechanisms, known as restriction factors, which stop viral replication in response to infection.

SARS-CoV-2 uses its surface envelope Spike glycoprotein (S-protein) to interact and gain access to host cells by biding to the Angiotensin-I converting enzyme-2 (ACE2) receptor. The S-protein-ACE2 interaction is the primary key to virus entry (**Figure 1**) (Shang et al., 2020; Jackson et al., 2022). The receptor binding domain located in the surface unit, S1, of the S protein of SARS-CoV-2, exhibits sequence homology with SARS-CoV (the agent of the SARS outbreak in 2003), which also uses ACE2 (Hoffmann et al., 2020; Zhou et al., 2020b; Hoffmann and Pöhlmann, 2022). Therefore, much of the knowledge about the S-protein adhesion process has been acquired throughout studies on SARS-CoV. However, alternative receptors have been identified for SARS-CoV-2.

One study identified the cellular proteins asialoglycoprotein receptor-1 (ASGR1) and Kringle Containing Transmembrane Protein 1 (KREMEN1) as alternative receptors for the S-protein from SARS-CoV-2 (Gu et al., 2022; Hoffmann and Pöhlmann, 2022). In that study, ASGR1 and KREMEN1 were expressed in an ACE2-deficient cell line resulting in SARS-CoV-2 but not SARS-CoV entry (Gu et al., 2022). This result was also supported in a mouse model infected with SARS-CoV-2, although this did reveal that entry *via* ASGR1 and KREMEM1 was generally less efficient than ACE2-dependent entry.

Blocking the CD147 receptor with a monoclonal antibody (Meplazumab) has been shown to inhibit SARS-CoV-2 from invading Vero E6 cells (Wang et al., 2020; Masre et al., 2021; Jackson et al., 2022). Also, human T cells with natural deficiency of ACE2 are infected by a SARS-CoV-2 pseudovirus, this infection can be inhibited explicitly by the Meplazumab anti-CD147 antibody (Wang et al., 2020). In murine models, viral loads were detectable in the lung of transgenic animals, which express human CD147, but not in wild-type mice (Wang et al., 2020). Establishing it as an alternative receptor for SARS-CoV-2.

Some other receptors have been presented as alternative receptors of SARS-CoV-2, such as Neuropilin-1 (NRP1) and Dipeptidyl peptidase 4 (DPP4). NRP1 was understood as another docking receptor to facilitate SARS-CoV-2 entry, and presents increased expression in biological samples from COVID-19 patients (Cantuti-Castelvetri et al., 2020; Masre et al., 2021; Jackson et al., 2022). The furin-cleaved S1 fragment from SARS-CoV-2 S-protein binds directly to NRP1, and blockage of this interaction by RNA interference or selective inhibitors reduces viral infection in cell culture (Daly et al., 2020). Regarding DPP4 (also known as CD26), it binds to the SARS-CoV-2 spike glycoprotein (Vankadari and Wilce, 2020), suggesting that SARS-CoV-2 may share the mode of entry through DPP4 with MERS-CoV.

However, it is not just the availability of receptors which determines the success of viral entry. For example, for SARS-CoV-2, a series of host factors also influence this process which is triggered by the virus, including interaction between the N-terminal domain (NTD) of the spike and lipid rafts, the affinity for ACE2, and some proteases involved in viral fusion and entry, namely furin, Transmembrane proteases serine 2 (TMPRSS2) and 4 (TMPRSS4), Cathepsin L1 (CTSL1) (Zang et al., 2020; Murgolo et al., 2021). Cleavage of the multibasic site (Arg-Arg-Ala-Arg) between the S1–S2 portions of the Spike protein is a prerequisite for cleavage of the S2′ site, and both cleavage events are essential to initiate the membrane-fusion process (Belouzard et al., 2012; Peacock et al., 2021; Zhang et al., 2021).

TMPRSS2 is present on the cell surface, and TMPRSS2-mediated S protein activation takes place at the plasma membrane, whereas cathepsin-mediated activation takes place in the endolysosome (**Figure 1**) (Shulla et al., 2011; Gomes et al., 2020; Shang et al., 2020; Zhang et al., 2021; Zhao et al., 2021). Therefore, depending on the entry route taken by SARS-CoV-2, the S2′ site is cleaved by different proteases. SARS-CoV-2 has two known entry pathways and can target either of them according to the cell type expression of TMPRSS2 (Koch et al., 2021). When the expression of TMPRSS2 is insufficient, the virus–ACE2 complex is internalised *via* clathrin-mediated endocytosis (Bayati et al., 2021). In the endolysosomes, cleavage by cathepsins occurs after acidification of the endolysosome environment, which triggers the fusion of the viral membrane with the endolysosome membrane (Jackson et al., 2022). In contrast, when entry occurs in the presence of TMPRSS2, cleavage occurs on the cell surface, triggering the fusion of the viral membrane with the plasma membrane (Jackson et al., 2022).

The expression of receptors in cell lines directly impacts the usage of entry pathways by SARS-CoV-2 and a main advantage of cell culture systems is that it allows direct visualization and quantification of the SARS-CoV-2 entry and replication processes. A study using the SARS-CoV-2 Wuhan 1 strain (B lineage) estimated the relative usage of entry pathways in different cell lines: in 293T cells expressing ACE2, the virus used nearly exclusively the Cathepsin B/L pathway, and in Vero expressing TMPRSS2 at approximately 65% of the time entry occurred *via* the TMPRSS2-mediated pathway (Padmanabhan et al., 2020). Similar results to those obtained in this Vero cell model were found in Hela cells that expressed both ACE2 and TMPRSS2. In Caco-2 cells, usage of the TMPRSS2 pathway hit around 85% and nearly 100% for Calu-3 cells (Padmanabhan et al., 2020).

However, it is not just the expression of receptors and proteases that dictate this dominant profile of entry pathway in each cell type. The emergence of new variants of SARS-CoV-2 showed that the mutations accumulated by the virus also influence the virus’s preferred entry pathway. For example, in an analysis performed by Padmanabhan and Dixit (2022 - Preprint) from the results of Hoffmann et al. (2022), who measured the extent of infection of cells *in vitro* by the Omicron variant (BA.1 sub-lineage) relative to other variants in different cell types, found that the Omicron pseudotyped virus entry was less efficient than the Wuhan 1 strain (B lineage) and Delta variant strains in Calu-3 and Caco-2 cell lines where the TMPRSS2 entry pathway is dominant for the Wuhan 1 strain. In parallel, the entry of the Omicron variant would be more efficient in HEK 293T and Vero E6 cells where the Cathepsin B/L entry pathway is dominant by the original B lineage (Hoffmann et al., 2022; Padmanabhan & Dixit, 2022 - Preprint). Entering the host cell alone is no guarantee of taking control of the cellular machinery, since cell susceptibility and permissibility to any given virus may vary from cell to cell, even when the cells are derived from the same tissue.

### Resistance, susceptibility, and permissibility

There are many cellular functions upon which viruses depend on when it comes to successfully infecting a cell. For a productive infection to be successful, cellular structures and molecular pathways must ensure all essential viral replication events, including adhesion, entry, uncoating, replication, assembly, and the release of new infective particles (Louten, 2016). For example, a cell without the specific receptor for a given virus is considered resistant to this virus. However, However, genetic engineering can reverse these misfortunes, and the gene encoding the cell receptor can be inserted into the cell’s genome that allows to express it on the cell surface (Ramirez et al., 2021).

In this context, a cell that allows the adhesion and entry of the virus is understood as being susceptible to the virus (Faisst, 1999). The presence of the functional receptor on the cell surface alone does not guarantee that this cell will support viral replication. Cells have developed certain defense mechanisms against viral infection, producing antiviral restriction factors which make the cell less permissive to viruses. Antiviral restriction factors are proteins produced by the host cell, which constitute a first line strategy to block viral replication and propagation, by interfering at critical steps of the viral replication cycle or triggering innate responses (Colomer-Lluch et al., 2018). This is well-exemplified in HIV-resistant cells due to the expression of numerous robust antiviral factors, such as the apolipoprotein B mRNA editing enzyme, catalytic subunit 3G (APOBEC3G) and the tripartite motif-containing protein 5 (TRIM5α). TRIM5α for example, binds to sensitive, incoming retroviruses *via* its C-terminal domain and recruits them to the proteasome where it is degraded (Huthoff and Towers, 2008).

Most antiviral resistance factors are Interferon Stimulated Genes (ISGs), in other words, genes that activate their expression after detecting interferons by cellular receptors (Schneider et al., 2014; Danziger et al., 2022). Binding interferon to their receptors triggers a series of signaling cascade in the cell to protect against an infection. ISGs encode functionally diverse gene products, including the antiviral effectors that antagonise distinct steps of viral life cycles (Schoggins, 2019; Danziger et al., 2022). As they depend on an interferon stimulus, induced resistance usually is not a concern for *in vitro* virology studies, as most cell lines used for viral production do not produce endogenous interferons. However, studies often use cells that express interferons or stimulate cells with interferon to better understand the mechanisms of resistance to viral infections.

Multiple studies have reported evidence implicating interferon as a key component in the host response to SARS-CoV-2. Several *in vitro* reports have highlighted that it effectively blocks SARS-CoV-2 infection when added to cell culture prior to the infection (Felgenhauer et al., 2020; Lokugamage et al., 2020). In parallel, transcriptome analyses of interferon-stimulated cell lines make it possible to identify ISGs and the resistance factors that restrict SARS-CoV-2 replication at different stages of the replication cycle (**Supplementary Table 1** and **Figure 1**) (Bruchez et al., 2020; Nchioua et al., 2020; Lee et al., 2021; Martin-Sancho et al., 2021; Danziger et al., 2022; Mac Kain et al., 2022). Lymphocyte Antigen 6 Family Member E (LY6E) has been identified as a restriction factor for SARS-CoV-2 entry, inhibiting viral Spike protein-mediated membrane fusion (Pfaender et al., 2020; Zhao et al., 2020b; Martin-Sancho et al., 2021).

Other factors have been identified as inhibiting SARS-CoV-2 RNA translation or replication. The Interferon Induced Protein with Tetratricopeptide Repeats 3 (IFIT3), Spermatogenesis Associated Serine Rich 2 Like (SPATS2L), DnaJ Heat Shock Protein Family (Hsp40) and Member C6 (DNAJC6) expression on HEK 293T cells leads to a significant decrease in SARS-CoV-2 RNA levels (Martin-Sancho et al., 2021). IFIT3 is known to prevent active viral RNA replication by detecting and sequestering single-stranded 50-ppp or 20 O-unmethylated RNA (Metz et al., 2013), while SPAT2SL is recruited to cytoplasmic stress granules sequestering viral RNA and reducing viral genome synthesis (Zhu et al., 2008; Miller, 2011).

Restriction factors that prevent viral release, such as the Bone Marrow Stromal Cell Antigen 2 (BST2), have been identified as potent inhibitors of SARS-CoV-2 release in HEK 293T cells (Martin-Sancho et al., 2021). BST2 inhibits the release of several enveloped viruses, such as HIV-1 and other CoVs, by tethering the virions to the cell surface or intracellular membranes (Neil et al., 2008; Van Damme et al., 2008; Wang et al., 2014; Taylor et al., 2015). The effect of this restriction factor is antagonized by SARS-CoV-2 Orf7 (Martin-Sancho et al., 2021).

Antagonizing innate host cell responses is a common strategy among viruses. The interferon pathway itself has been described as being antagonized by various mechanisms involving the SARS-CoV-2 proteins ORF3b, ORF8, ORF9b ORF6, Nsp15 and Spike (Ribero et al., 2020; Zinzula, 2021; Freitas et al., 2022). In conclusion, viral replication in an infected cell is the result of complex interactions between the host and viral proteins. Conversely, cells that allow post-entry steps, such as uncoating, replication, assembly, and release, are then understood to be virus-permissive cells (Faisst, 1999). Therefore, in most virus assays, the aim is to find cell models that are both susceptible and permissive to the virus being studied.

### Types of infection

When choosing to work with cells that are both susceptible and permissive to a virus, what is being sought is a complete infection, where the virus infects, takes control of the cellular machinery, expresses its structural and non-structural components, and ensures the assembly and release of infectious particles at the end of the cycle. However, infected cells can also undergo abortive infections. Thus, although the cells are infected with a virus, they do not produce any progeny virus due to this infection (Cohen et al., 2019). Abortive viral infections are frequently observed even during the infection of susceptible and permissive cell types, however, some single-cell studies of viral infections have suggested that even in permissive cells, around 40% of the infected cells do not produce progeny viruses (Combe et al., 2015; Heldt et al., 2015). This suggests that abortive infection is a common outcome for many viral infections.

Abortive SARS-CoV-2 infections have been described for different cells. In primary cell culture, such as the primary human umbilical vein endothelial cells (HUVECs), human microvascular endothelial cells from the lung (HMVEC-L), and human primary lung microvascular endothelial cells (HL-mECs), increased nucleocapsid protein expression of SARS-CoV-2 has been detected, but without generating infectious progeny (Caccuri et al., 2020; Schimmel et al., 2021). Abortive SARS-CoV-2 in T cells (Flemming, 2021; Swadling et al., 2021) and brain endothelial cells (Bauer et al., 2022). However, the consequences of abortive infections on the pathophysiology of SARS-CoV-2 are still uncertain. Therefore, here we will focus on cells that allow effective SARS-CoV-2 infection, characterized by the production of infectious viral particles.

### Cytopathic effect

The observation of cytopathic effects (CPE) is crucial in virology. CPE refers to structural changes in the host cells due to viral invasion and replication. Some viruses cause characteristic morphological alterations in the infected cells, such as rounding and detaching, fusion with adjacent cells to form syncytia, and the appearance of nuclear or cytoplasmic inclusion bodies (Faisst, 1999; De Souza et al., 2020). Observing the CPE is essential for virologists who isolate and identify viruses.

Combining a virus with a permissive cell that presents CPE is desired, since the presence of CPE can provide the researcher with indications about the course of infection. Usually a peak in visualizing CPE on the cell monolayer indicates the best time to collect viral production, although it may be desired to determine the protection of these cells in the antiviral assays and serological evaluation (Faisst, 1999), for example. A cytopathic effect also enables relatively simple assays for quantifying infectious particles, known as viral titration assays, which can be based on, for example, counting plaque forming units (PFU) or observing CPEs to determine Median Tissue Culture Infectious Dose (TCID50) (Faisst, 1999; Payne, 2017). Although these previously mentioned viral quantification methods are widely used, other methods have been developed for viruses that cannot find the virus-cell pair that results in CPE, such as focus-forming and hemagglutination assays or even enzyme-linked immunosorbent assays (ELISA), a cell-based indirect immunofluorescence assay, which involves antibody-based approaches (Payne, 2017).

The lighter CPE typically seen for SARS-CoV-2 is present in cells derived from the kidneys of the African green monkey (*Chlorocebus sabaeus*) and rhesus macaque (*Macaca mulatta*). In Vero/Vero E6, MA1040, and BGM cells, cell rounding can be observed 48 hours after the infection is initiated in an asynchronous infection cycle (Wurtz et al., 2021). However, other cell lines can present high SARS-CoV-2 production levels, without presenting evident CPE, as do Caco-2 cells.

## Isolating and producing SARS-CoV-2

Isolating viruses is still a sensitive and essential method for virologic diagnosis and has long served as the “gold standard” for virus detection (Hosoya et al., 1998; Mizuta et al., 2003; Leland and Ginocchio, 2007). Currently, the isolation of viruses has lost ground to molecular detection methods, although especially in the field of virus research having a viral strain isolated allows researchers to study the biological aspects of a specific virus, such as the replication cycles, the entry process, and the receptors used by the virus, for example.

This isolated virus can also enable the production of antigens or can be employed in antiviral susceptibility screening tests (Leland and Ginocchio, 2007). However, this posterior assay requires a well-characterised viral stock produced in cell culture, ideally with minimal passaging to reduce the rise of genetic sub-populations and heterogenicity of phenotypes of the virus stock (Baczenas et al., 2021). Therefore, this virus stock will serve as a seed stock which will be propagated to make more extensive stocks.

When isolating a virus in cell culture, the experimenter often looks for permissive cells that present CPE, since the aspect of the CPE is often characteristic of certain viruses in each cell and indicates a successful infection (Faisst, 1999). In addition, when producing a sizeable viral stock, high viral titers are often sought (Leland and Ginocchio, 2007), in other words, a cell with a high production of infective viral particles.

### *Verda reno* E6 cells (Vero E6)

Vero cells were established from kidney tissue sampled from an African green monkey (*C. sabaeus*) (Ammerman et al., 2008; Naoki et al., 2014). They originated from a primary culture initiated in March 1962 by a group from Chiba University in Japan. Over the months of serial passages of these cells, the researchers obtained a series of sub-lines, one of which was chosen as the standard Vero cell line (Ammerman et al., 2008; Naoki et al., 2014).

This cell lineage was widely distributed among research laboratories and has become one of the most common mammalian immortalized cell lines used in research (Ammerman et al., 2008). In the field of virology, these cells have gained prominence for being susceptible to a wide range of viruses, such as simian polyomavirus SV-40, rubella virus, arboviruses, adenoviruses, H5N1 influenza virus, Ebola hemorrhagic fever virus 19, SARS-CoV, and MERS-CoV (Ellis et al., 1979; Horimoto and Kawaoka, 2006; Zaki et al., 2012; Kiesslich and Kamen, 2020). Vero cells are also widely used in vaccine production as safe cell substrates for antigen production (Barrett et al., 2009).

The Vero E6 clone was obtained by using the dilution method into microtiter plates in 1979 by PJ Price (Yamate et al., 2005). The main characteristic of Vero E6 is that it E6 exhibits contact inhibition after forming a monolayer; therefore, it helps growing slow-replicating viruses. Since 2003, Vero E6 cells have also been used extensively for SARS-CoV-like virus research and cell-culture-based infection models by many laboratories as it supports viral replication to high titers. This high susceptibility could be related to the high expression level of the ACE-2 receptor (Gillim-Ross et al., 2004), which is used by both SARS-CoV-2 and SARS-CoV (Hoffmann et al., 2020) and/or their lack of ability to produce interferon (De Clercq et al., 1973; Emeny and Morgan, 1979).

**Figure 2:**
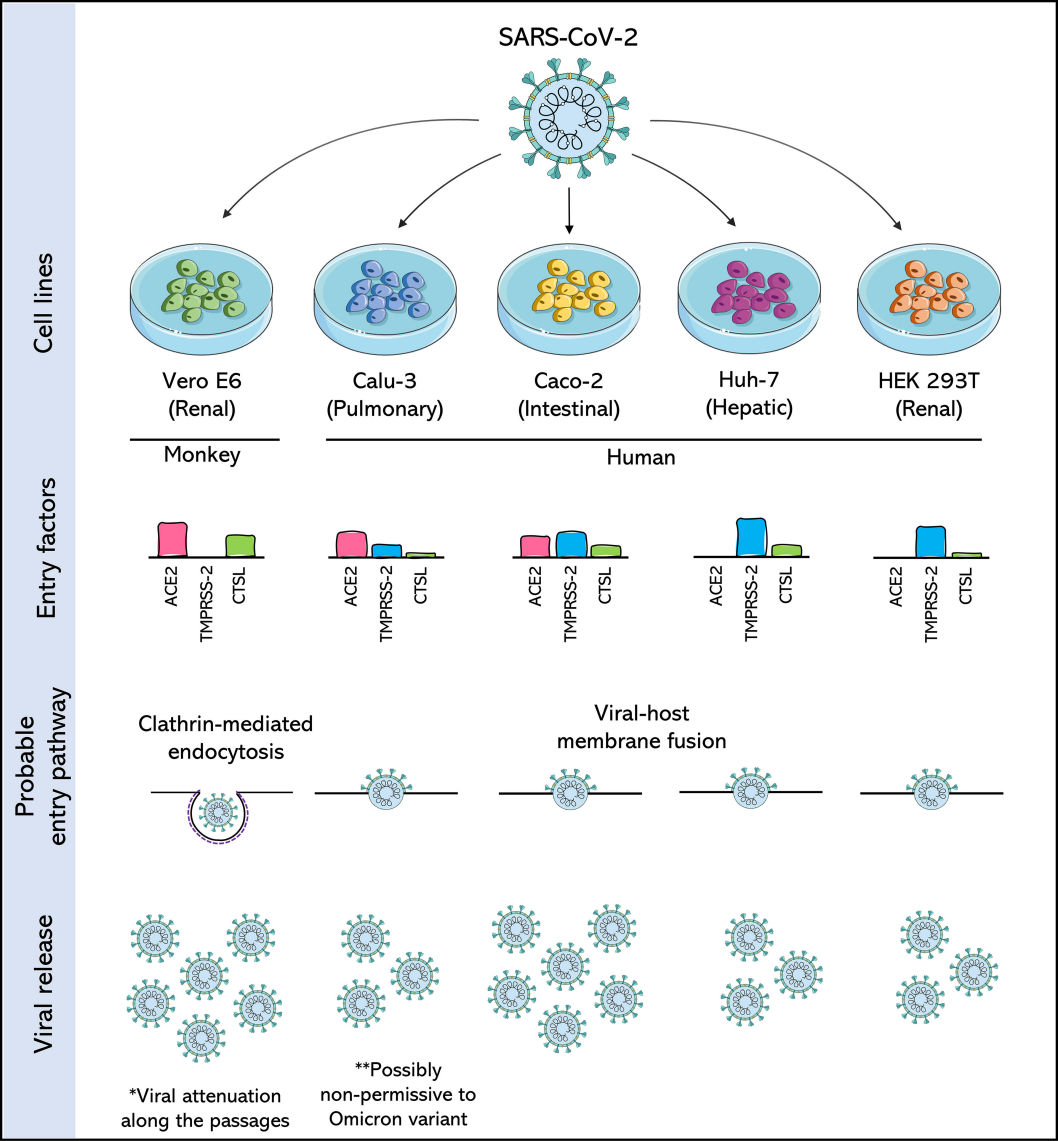
Comparison of SARS-CoV-2 permissive cell lines. Four human cell lines: Calu-3 (Pulmonary), Caco-2 (Intestinal), Huh-7 (Hepatic) and HEK 293T (Renal), are compared with each other and with the Vero E6 cell, a cell derived from the African green monkey kidney, and widely used in the isolation and production of SARS-CoV-2. Cells are compared for the expression of three important entry factors used by SARS-CoV-2: angiotensin-converting enzyme 2 receptor (ACE2), transmembrane protease serine 2 (TMPRSS2) and the lysosomal protease cathepsin L (CTSL), based on data from Murgolo et al., 2021; Saccon et al., 2021 and Shuai et al., 2020. The expression of these factors dictates the entry pathway used by SARS-CoV-2. The release of SARS-CoV-2 viral particles is also compared. The asterisks draw attention to likely consequences of producing SARS-CoV-2 in these cells.

Vero E6 cells are, therefore, highly susceptible, and permissive cells to SARS-CoV-2, being able to be used both in the isolation and production of viral stocks due to the abundance of receptors and because they present characteristic CPE (cell rounding, detaching and lysis) as well as allow the recovery of high titers of SARS-CoV-2 (**Figure 2**). Furthermore, studies also point to these cells as a suitable basis for performing the initial screening of antiviral compounds, which makes it possible to select the most promising hits for in-depth mechanistic studies in other cells (Ogando et al., 2020). The high viral titers obtained in Vero E6 cells can also be influenced by the medium in which the cells are grown, as demonstrated by cultivating these cells in Physiological Plasmax Medium, which, when infected by different RNA viruses, including SARS-CoV-2, had lower viral release rates compared to the cells cultivated under Dulbecco’s Modified Eagle Medium (DMEM) (Golikov et al., 2022).

However, studies have shown that SARS-CoV-2 adapts rapidly to passages in Vero E6 cells (Lamers et al., 2021; Pyke et al., 2021). For example, SARS-CoV-2 exhibited remarkable phenotypic variation in the plate assays within a few passages after its isolation from the clinical samples (Ogando et al., 2020). This change in phenotype has been associated with the emergence of mutations near the so-called furin-like S1/S2 cleavage site, which triggers its inactivation and provides a selective advantage over SARS-CoV-2 passages in Vero E6 (Ogando et al., 2020; Peacock et al., 2021; Sasaki et al., 2021). This, in turn, leads to favoring an entry pathway in that cell with low expression of TMPRSS2. It was also reported that the conversion of procathepsin L to cathepsin L (CTSL) is significantly higher in Vero cells than in other susceptible cell lines, such as Calu-3, Caco-2, and Huh-7 (**Figure 2**) (Koch et al., 2021; Zhao et al., 2021).

This information raises issues around the number of passages that are made in that cell, as they can lead to attenuation of the virus, mainly if later experiments are to be carried out in other cells and if they require a TMPRSS2-dependent entry route. This attenuation can be easily avoided by adopting an engineered VeroE6/TMPRSS2 cell line, understood as highly susceptible to SARS-CoV-2 (Matsuyama et al., 2020). Although most studies using SARS-CoV-2 employ the Vero E6 sub-lineage, other Vero lineages have also been shown to be permissive to the virus, such as VERO 81 and VERO SLAM, this last one transfected with a plasmid encoding the gene for the human signaling lymphocytic activation molecule (SLAM) molecule (Wurtz et al., 2021).

It must, however, be considered that Vero cells are cells derived from African green monkey tissue and, therefore, a non-human primate (NHP). Although it is a species phylogenetically close to humans, with low ACE2 polymorphism compared to human ACE2, this may represent a limitation for studying a human virus such as SARS-CoV-2. This means a restriction on the information recovered during drug screening, especially if the drugs are designed to be metabolised in human cells (Pruijssers et al., 2020; Ramirez et al., 2021). Therefore, many assays seek to employ cells derived from human tissues to prevent the isolates from suffering attenuation in cells of other species and especially for drug screening.

## Human cell lines

### Cultured human airway epithelial cells (Calu-3)

As SARS-CoV-2 was initially understood to be a virus that triggers diseases in the lower respiratory tract (Murgolo et al., 2021), the search for a human cell derived from lung tissue that was permissive to this virus became essential for the *in vitro* study of the disease, its pathogenicity and especially anti-SARS-CoV-2 drug screening.

Calu-3 cells are a human lung adenocarcinoma cell line isolated in 1975 from the pleural effusion of a 25-year-old Caucasian male (Fogh et al., 1977). Calu-3 is commonly used in cancer research and drug development (Zhu et al., 2010) and was understood as a highly permissive cell to SARS-CoV (Tseng et al., 2005). However, during the previous epidemic in 2003, when it was initially challenging to establish infection models in cells derived from human lungs, this compromised studies on the pathogenesis of the SARS-CoV, which, as is the case in SARS-CoV-2, mainly causes manifestations in the respiratory tract (Tseng et al., 2005; Grossegesse et al., 2022).

The knowledge already established from the 2003 SARS-CoV outbreak was revisited with the emergence of SARS-CoV-2 in 2019, and Calu-3 cells were perceived as permissive cells for both viruses. SARS-CoV-2 grew faster and at a higher titer than SARS-CoV in Calu3 cells (Chu et al., 2020). However, comparative studies demonstrate that the production of SARS-CoV-2 by Calu-3 is usually lower than that obtained in Vero E6 cells (**Figure 2**), and, unlike these latter cells, Calu-3 cells do not show CPE when infected with SARS-CoV-2 (Chu et al., 2020; Park et al., 2021; Wurtz et al., 2021; de Souza et al., 2022).

In addition to these two disadvantages of using Calu-3 Cells, a third can also be mentioned. Calu-3 cells grow more slowly than Vero cells (Baczenas et al., 2021), but this does not mean they are inferior to Vero cells. Instead, this is because, unlike in Vero cells, the furin cleavage site appears to be preserved in SARS-CoV-2 produced in Calu-3 cells, rather than promoting the attenuation typically observed during passages in Vero cells (Baczenas et al., 2021; Lamers et al., 2021; Mykytyn et al., 2021a).

This type of feature makes Calu-3 cells interesting for SARS-CoV-2 studies, particularly if this stock is being produced for infections in animal models, as Calu-3-derived virus stocks remained pathogenic in hamsters, and the Calu-3-specific variants were maintained (Baczenas et al., 2021). In addition, these cells are also widely used in drug screening studies against SARS-CoV-2 (Dittmar et al., 2021; Tao et al., 2021; Coimbra et al., 2022).

However, with the emergence of many variants of SARS-CoV-2, several of them being classified as variants of concern by the WHO, it is unclear whether mutations acquired by the virus within the population can impact viral fitness (Harvey et al., 2021). For example, for the Omicron (B.1.1.529) variant of concern, mounting evidence, mainly from animal studies, suggests that Omicron BA.1 sub-lineage does not readily multiply in lung tissue (Abdelnabi et al., 2022; Halfmann et al., 2022; McMahan et al., 2022). This could impact the production of this and other new variants by Calu-3 cells, which would require a new adaptation of the isolates to the cells through different passages, which consequently would distance them from the original isolate and the circulating variant.

### Cancer coli 2 cells (Caco-2)

Although SARS-CoV-2 primarily affects the respiratory system, increasing evidence suggests that this virus may have gastrointestinal manifestations. The SARS-CoV-2 genome was previously found in gastric, rectal, and duodenal mucosa samples, suggesting that the digestive system is a potential source of viral transmission (Arslan et al., 2020; Kipkorir et al., 2020; Zhang et al., 2020). SARS-CoV-2 replicates in gastrointestinal cells *in vivo* and is frequently detected in faeces (Chen et al., 2020; Cheung et al., 2020; Lee et al., 2020b; Wu et al., 2020; Xiao et al., 2020), providing evidence that SARS-CoV-2 can also infect intestinal cells

Caco-2 cells were established in 1977 from a colorectal adenocarcinoma taken from a 72-year-old Caucasian man using the explant culture technique (Fogh et al., 1977). The Caco-2 cell line demonstrated morphological and biochemical characteristics of small intestine enterocytes (Hidalgo et al., 1989) and has been widely used to study infection first with SARS-CoV and now with SARS-CoV-2 (Cinatl et al., 2004; Bojkova et al., 2020). These cells have become, along with Calu-3 cells, the main human cell lines explored in the *in vitro* studies of SARS-CoV-2.

Compared to Calu-3 cells, Caco-2 cells are suggested to have lower expression of the ACE2 receptor but higher expression of TMPRSS2 (**Figure 2**) (Shuai et al., 2020; Saccon et al., 2021). Although no assays have demonstrated that the furin cleavage site is maintained in SARS-CoV-2 produced in Caco-2, as is observed for the viral stocks produced in Calu-3, the high expression of TMPRSS2 is a strong indication that the attenuation observed by passages in Vero cells would not occur in this cell line.

Continuing with the comparison of Caco-2 cells and Calu-3 cells, one proteomics study pointed out that Caco-2 cells have 177 proteins which are differentially expressed during SARS-CoV-2 infection, while Calu-3 has more the 6,000 such proteins (Saccon et al., 2021). Yet another study showed that among the five cytokines/chemokines evaluated, SARS-CoV-2 infection leads to increased expression only of Interferon gamma-induced protein 10 (IP-10), but not of Tumor Necrosis Factor-alpha, RANTES (regulated on activation, normal T cell expressed and secreted), Interleukin 6, or Interleukin 8 in Caco-2 cells (Shuai et al., 2020). No relative increase in these cytokines/chemokines was observed in Calu-3 cells infected with SARS-CoV-2 (Shuai et al., 2020). Other authors suggest that the absence of CPE in Caco-2 could be due to the weak pro-inflammatory response triggered by SARS-CoV-2 (Zupin et al., 2022).

The absence of CPE in Caco-2 cells is suggested at least for low multiplicity of infections (MOIs) (Chu et al., 2020; Saccon et al., 2021; Wurtz et al., 2021; Mautner et al., 2022; Zupin et al., 2022) and this is its main disadvantage compared to Vero E6 cells. Furthermore, unlike Calu-3 cells, SARS-CoV-2 viral production by Caco-2 cells typically reaches levels similar to or higher than those seen in Vero E6 cells (**Figure 2**) (Chu et al., 2020; Koch et al., 2021; Saccon et al., 2021; de Souza et al., 2022). These characteristics make Caco-2 cells a good choice of human cells for SARS-CoV-2 assays.

### Human hepatoma 7 cells (HuH-7)

The HuH-7 cell line was established from an already well-differentiated hepatocyte derived from a cellular carcinoma cell line originally collected from a liver tumour from a 57-year-old Japanese man. The line was established by Nakabayashi et al. (1982), who found that this epithelial-like cell replicated continuously in a chemically defined medium when the medium was supplemented with Na_2_SeO_3_ (Kawamoto et al., 2020). Huh-7 is highly susceptible to the hepatitis C virus (HCV) and is, therefore, often used for the *in vitro* production of infectious HCV particles and in anti-HCV drug assays in a replicon-based system (Liu et al., 2014).

Huh-7 cells are permissive to different human coronaviruses, such as CoV 229E, CoV OC43, MERS-CoV, and SARS-CoV (Ois Freymuth et al., 2005; de Wilde et al., 2013). This cell also exemplifies how different viruses, even if they belong to the same family, can have different patterns of cytopathic effect in the same cell. For example, using Huh-7 as an infection model, MERS-CoV was highly cytopathic. At the same time, SARS-CoV presented a delayed CPE, and no CPE was observed for SARS-CoV-2 using the same infectious dose for the three viruses (Chen et al., 2021).

Unlike other cells, despite Huh-7 is permissive to SARS-CoV-2, co-expression analyses of ACE2 and TMPRSS2 revealed that Huh-7 cells strongly expressed TMPRSS2 but lacked ACE2 expression (**Figure 2**), which was understood as an indication that each receptor plays an individual role in aiding the infection (Saccon et al., 2021). A post-infection proteomics study pointed out that there is no change in the global protein abundance in Huh-7 infected with SARS-CoV-2, with only four proteins being differentially expressed (Saccon et al., 2021). In this same cellular model, a type I interferon signature induced by SARS-CoV-2 infection distinct from that with other coronaviruses such as SARS-CoV and MERS-CoV, was also observed (Chen et al., 2021).

Although Huh-7 cells are permissive to SARS-CoV-2, viral replication in these cells is considered to be moderate (**Figure 2**) (Chu et al., 2020), possibly due to the aforementioned factors regarding receptor expression, interferon signature, and protein expression. However, retaining a cell of liver origin in culture for SARS-CoV-2 studies is particularly interesting, as the liver has been identified as one of the main target organs in cases of COVID-19, and the rate of incidence of liver injury in these patients is 14%–53% (Zhou et al., 2021a; Wanner et al., 2022). Furthermore, Huh7, like many previously cited cell lines, is an immortalised cancer cell line that may not be physiologically representative of human tissue.

### Human embryonic kidney 293T cells (HEK 293T)

The HEK 293T is an important sub-lineage of the HEK 293 cell line, differing from it in that it contains the temperature-sensitive mutant Simian Vacuolating Virus 40 (SV40) T-antigen that allows episomal replication of transfected plasmids containing the SV40 origin of replication. It was introduced by Michele Calos’s lab at Stanford in 1987 (Rio et al., 1985; Dubridge et al., 1987). This modification made these cells particularly popular for producing recombinant proteins and retroviruses (Pear et al., 1993).

Like Vero cells, HEK 293 cells, from which HEK 293T cells were derived, are kidney-derived epithelial cells. They were established by Graham et al. (1977) for the transformation of primary human embryonic kidney (HEK) cells obtained from a spontaneously miscarried or aborted female following exposure to sheared fragments of human adenovirus type 5 DNA (Graham et al., 1977; Shaw et al., 2002).

The HEK 293T cells strongly expresses TMPRSS2 but lack ACE2 expression (**Figure 2**). At the same time, the post-infection proteomics studies have not found any change in the global protein abundance in HEK 293T infected with SARS-CoV-2, results that are similar to those obtained for Huh-7 cells (Saccon et al., 2021). The results of these two cells are also similar for the production of SARS-CoV-2 in that both are understood as low-level virus production compared with Caco-2 (**Figure 2**) (Chu et al., 2020; Saccon et al., 2021). As with the other human cells mentioned above, there is no cytopathic effect on HEK 293T cells after SARS-CoV-2 infection (Chu et al., 2020).

HEK 293 cells appear to produce only abortive SARS-CoV-2 infections; therefore, only HEK 293T cells should be used in SARS-CoV-2 studies (Harcourt et al., 2020; Modrof et al., 2020). HEK 293T cells are embryo cells and, due to differences in receptor expression and viral production, it is difficult to compare these human kidney cells with monkey kidney cells (Vero cells). Moreover, there are doubts as to whether they can be minimally representative in studies of the renal pathology of SARS-CoV-2.

Nevertheless, HEK 293T cells appear to be valuable tools for studies evaluating the impact of specific protein expression on viral replication once these cells can be transfected with high efficiency and support productive replication of SARS-CoV-2. One study that evaluated antiviral restriction factors at various stages in the SARS-CoV-2 replication cycle was conducted in HEK 293T cells transduced with lentiviruses carrying the gene previously identified as a potential restriction factor (**Supplementary Table 1**) (Martin-Sancho et al., 2021). This type of approach is essential for understanding virus-host interactions.

## Other permissive mammalian cell lines

With the emergence of SARS-CoV-2, some studies were dedicated to testing the susceptibility of commonly used laboratory cell lines, and therefore, immortalized cells, to the new coronavirus (Chu et al., 2020; Saccon et al., 2021; Wang et al., 2021; Wurtz et al., 2021). This was a way to quickly present cells that could be used for the isolation and propagation of SARS-CoV-2 and to define the first line of experimental models to study the pathogenicity and perform antiviral assays on the emerging virus (Saccon et al., 2021). In addition to the human cells presented above, many mammalian cells were identified as at least susceptible to SARS-CoV-2 infection (**Table 1**).

**Table 1:** SARS-CoV-2 permissive mammalian cells lines. Commonly used laboratory mammalian cell lines that can support SARS-CoV-2 replication with infective particles release at the end of the cycle.

Cell Line	Organism	Tissue	Morphology	Cytophatic Effects	References
Caco-2	Human (*Homo sapiens*)	Colorectal adenocarcinoma	Epithelial	Absent	Chu et al., 2020; Saccon et al., 2021; Wurtz et al., 2021
Calu-3		Lung adenocarcinoma	Epithelial	Absent	Chu et al., 2020; Saccon et al., 2021
HEK 293T		Kidney	Epithelial	Absent	Chu et al., 2020; Saccon et al., 2021
Huh-7		Liver tumour	Epithelial	Absent	Chu et al., 2020; Saccon et al., 2021
U251		Glioblastoma	Pleomorphic/astrocytoid	Absent	Chu et al., 2020
BGMK	African green monkey (*Chlorocebus sabaeus*)	Kidney	Epithelial	Cell rounding and detaching	Wang et al, 2021; Wurtz et al., 2021
MA-104			Epithelial	Cell rounding and detaching	Wurtz et al., 2021
Vero E6			Epithelial	Cell rounding, detaching and lysis	Wang et al, 2021; Wurtz et al., 2021
CV-1			Fibroblast	Cell rounding and detaching	Wurtz et al., 2021
LLC-MK2	Rhesus macaque (*Macaca mulatta*)	Kidney	Epithelial	Absent	Wang et al, 2021; Wurtz et al., 2021
RhMK			Epithelial	Cell rounding and detaching	Wang et al, 2021; Wurtz et al., 2021
CRFK	Cat (*Felis catus*)	Kidney	Epithelial	Absent	Wang et al., 2021. Chu et al., 2020
PK-15	Pig (*Sus scrofa*)	Kidney	Epithelial	Cell rounding and detaching	Chu et al., 2020; Meekins et al., 2020; Lee et al., 2020b
ST		Testicle	Fibroblast	Cell rounding and detaching	Meekins et al., 2020
RK-13	Rabbit (*Oryctolagus cuniculus*)	Kidney	Epithelial	Absent	Chu et al., 2020

Cells derived from non-human primate kidneys are often thought to be cells which are susceptible to SARS-CoV-2 infection. Buffalo green monkey kidney cells (BGMK) and Cellosaurus cell line MA-104, both epithelial cells, and Cellosaurus cell line 1 (CV-1), with fibroblast morphology, are all derived from African green monkey kidneys (*C. sabaeus*) and appear to support the SARS-CoV-2 replication cycle (Wang et al., 2021; Wurtz et al., 2021). Rhesus macaque kidney epithelial LLC-MK2 and RhMK cells are also suggested to be permissive cells for SARS-CoV-2 (Wang et al., 2021; Wurtz et al., 2021).

In addition to cells from non-human primates, cell lines derived from cats, rabbits and pig kidneys has also been identified with a limited permissibility to SARS-CoV-2 infection (**Table 1**) (Chu et al., 2020; Wang et al., 2021). The Crandell-Rees Feline Kidney Cell (CRFK), isolated in 1973 and generally used in the production of viruses used in vaccines for felines, was recently identified as having a mesenchymal phenotype, in contrast to the previous characterisation of epithelial cells (Lawsan, 2019; Lappin, 2005; Crandell, 1973). In addition, the transmission of SARS-CoV-2 between cat owners and felines and from an infected cat to naive felines has been previously presented and may explain the susceptibility of cat cells to the virus (Bosco-Lauth, 2020; Patterson, 2020).

The RK13 cell line was obtained from the kidney of a five-week-old rabbit and is commonly used for viral isolation, as the RK13 cells have proven to be permissible to infection by the herpes simplex virus, pseudorabies virus, vaccinia virus, rabbitpox virus, simian adenoviruses, rubella virus and SARS-CoV (Coombs et al., 1961; Fogel and Plotkin, 1969; Kaye et al., 2006). Susceptibility to SARS-CoV-2 was suggested by Chu et al. (2020). Rabbits are widely used as experimental animals in the laboratory (Thomas et al., 2012). The susceptibility of rabbits to SARS-CoV-2 has been previously demonstrated with the detection of infectious viruses from the nose and throat upon experimental viral inoculation (Mykytyn et al., 2021b).They may, therefore, represent precious models for the study of SARS-CoV-2.

In contrast, pigs inoculated with SARS-CoV-2 by a broader number of viral inoculation routes, did not show any productive infection (Meekins et al., 2020; Vergara-Alert et al., 2021), even though they presented seroconversion and the presence of neutralising antibodies 22 days post-infection when inoculated by parenteral routes (Vergara-Alert et al., 2021). The immune response of these animals was indicated as a key factor for the protection of these animals against COVID-19. In porcine primary respiratory epithelial cells, self-limiting SARS-CoV-2 replication was associated with higher rates of apoptosis in infected cells (Nelli et al., 2021). However, in swine testicle (ST) and kidney (PK-15) cell lines, it was possible to established SARS-CoV-2 infections in which CPE was visualised after two and four passages, respectively (Chu et al., 2020; Lee et al., 2020b; Meekins et al., 2020; Schlottau et al., 2020).

Unfortunately, neither the non-human primate cells nor the other mammalians cell lines have been thoroughly characterized from the perspective of use for studies with SARS-CoV-2. There is no information about the abundance of its receptors, and little is known about the virus replication rates in these cells compared to human cells or even Vero cells. Wurtz et al. (2021) presented viral titers measured by TCID50 that indicate that those obtained with BGMK and MA-104 cells are between three and ten times higher than those obtained by Vero E6 cells approaches for two distinct isolates of SARS-CoV-2 at seven days post-infection, and they observed evident CPE 48 hours post-infection (**Table 1**). The TCID50/mL results published by Wang et al. (2021) also point out viral productions which are higher or similar to Vero E6 cells for BGMK and CV-1 cells, while those with rhesus macaque (*M. mulatta*) kidney epithelial cells LLC-MK2 and RhMK are usually lower than those with Vero E6.

These studies employing SARS-CoV-2 against a range of cell lines commonly used in research laboratories (Wurtz et al., 2021 and Wang et al., 2021) also generate a great deal of information about cells that are not susceptible to SARS-CoV-2. These data are presented in **Supplementary Table 2** to prevent strains from being repeatedly tested against this virus or being erroneously used in studies.

## Overcoming SARS-CoV-2 culture restrictions

This SARS-CoV-2 not-permissive rage of immortalized cells has become a challenge for developing these virus studies. In addition to many cells not allowing the virus to replicate, many of the established human cell culture lines have low-level SARS-CoV-2 production levels (Takayama, 2020). However, these drawbacks can be overcome by applying biotechnology tools to design cells that can, conveniently, be explicitly used for SARS-CoV-2.

**Figure 3:**
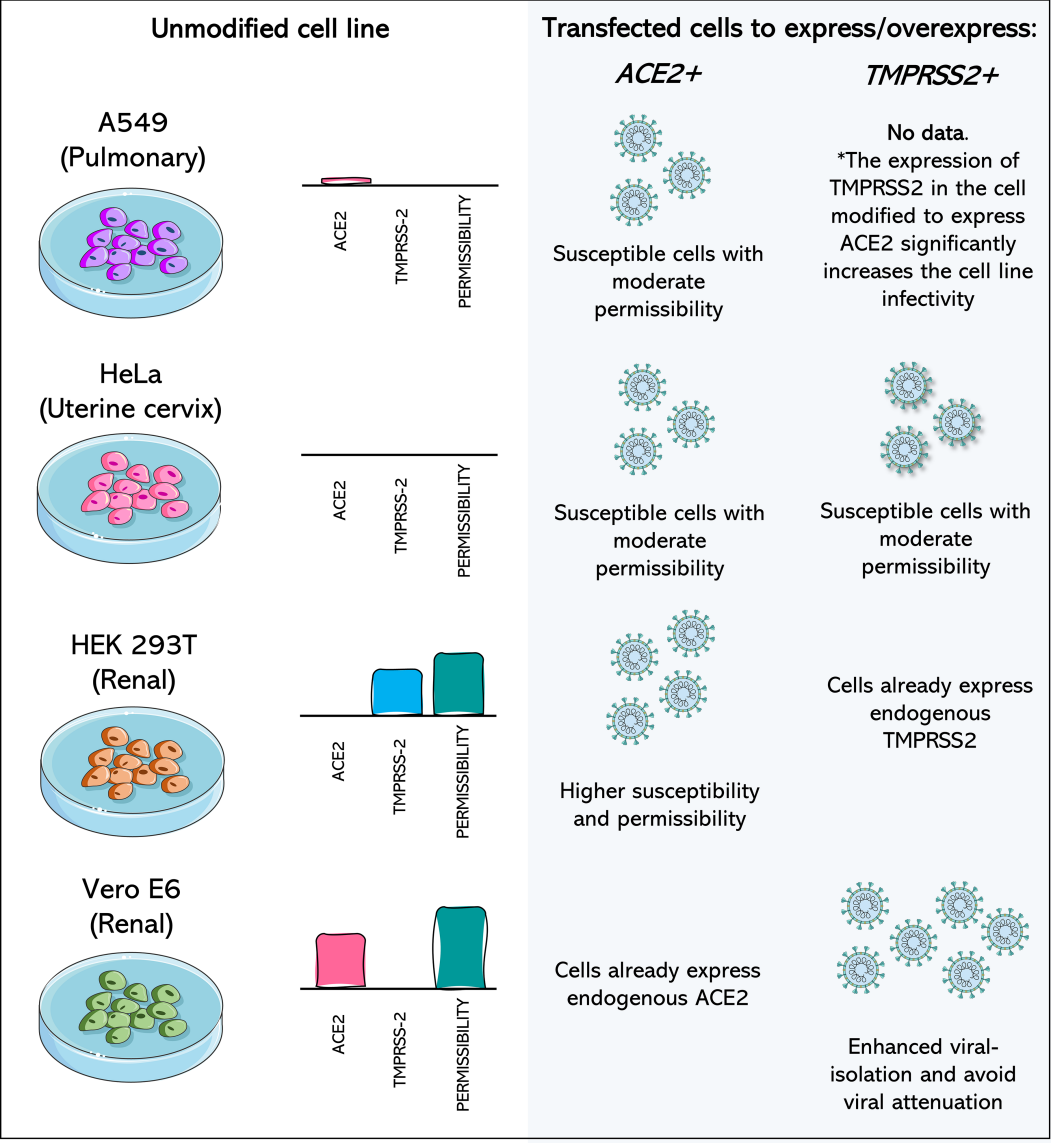
Cell lines transfected to express or overexpress ACE2 and TMPRSS2 show increased isolation of, susceptibility to, and permissibility for SARS-CoV-2. A549 (Pulmonary) cell line has poor expression of ACE2 and no expression of TMPRSS2, being considered a non-permissive cell to SARS-CoV-2. When ACE2 or both components are expressed after transfection of the cell, it becomes moderately permissive and susceptible to SARS-CoV-2. HeLa cells (Uterine cervix) are not susceptible to SARS-CoV-2 infection, as they do not express the viral receptors. Transfection of ACE2 and TMPRSS2 leads to susceptibility and moderate permissibility. In susceptible cells, such as Vero E6 monkey cells and HEK 293T and human renal cells, transfection with the receptor which they do not express endogenously leads to greater cell permeability. Vero E6/TMPRSS2+ cells have higher rates of SARS-CoV-2 isolation and potentially prevent attenuation of the virus. Data concerning the expression profile of each cell were recovered from Vectorbuilder; Murgolo et al., 2021; Saccon et al., 2021; Shuai et al., 2020 and Hoffmann et al., 2020. The asterisk (*) draws attention to the fact that no data was found about the impact of the expression of only TMPRSS2 in A549 cells, but the combination of TMPRSS2 expression in A549 cells already modified to overexpress ACE2 leads to increased infectivity.

Regarding cell susceptibility, cells were widely developed that expressed or overexpressed the receptors used by the virus for adhesion and penetration. For example, the lung adenocarcinoma cell line A549 was previously understood to be a cell which was not susceptible to SARS-CoV-2 (Chu et al., 2020; Wurtz et al., 2021), but the overexpression of human ACE2 in this cell line makes infection with SARS-CoV-2 possible (**Figure 3**) (Weston et al., 2020; Xie et al., 2020). The A549 cells overexpressing ACE2 has been shown to be valuable tools in anti-SARS-Cov-2 drug screening (Plaze et al., 2021; Pyrć et al., 2021; Napolitano et al., 2022). A new report of transduced A549 subclones selected to express more ACE2, and TMPRSS2 transcripts than existing commercial A549 cells engineered to express ACE2 and TMPRSS2 highlights its increased susceptibility, including to the Delta and Omicron variants, and its potential as a drug-inhibition cellular model (Chang et al., 2022).

Commercial HeLa-bases (derived from cervical cancer cells (Scherer et al., 1953)) and HEK 293T-based cell lines that are genetically modified to express ACE2, TMPRSS2, or both are currently available (**Figure 3**) (Vectorbuilder). HeLa-based cell lines have zero expression of endogenous TMPRSS2, and both HeLa and 293T-based cell lines do not express endogenous ACE2 (Vectorbuilder; Hoffmann et al., 2020). Combining cells that usually are not susceptible to a virus but that now express one or the other receptor, or both, is a valuable tool for understanding the role of each receptor in the viral entry process.

In addition to non-susceptible cells, the expression of additional receptors/co-receptors in cells known to be permissive to SARS-CoV-2 may represent a strategy to increase susceptibility and the rate of isolation. It has been well reported in TMPRSS2-expressing Vero E6 Cells, which were highly permissive to SARS-CoV-2 and other coronaviruses after this modification (Matsuyama et al., 2020). This leads not only to an increase in viral isolation rates but may also represent a way of overcoming the main limitation of these cells (**Figure 3**), which is the rapid attenuation of the virus (Sasaki et al., 2021).

With the emergence/selection of new variants and subvariants, this approach using the same cell modified to express one or more receptors becomes particularly interesting for investigating the impact of the accumulated mutations in the new variants/subvariants in viral invasion and pathogenesis. For example, the Omicron (B.1.1.529) BA.1 variant first identified in South Africa and Botswana rapidly spread globally, being classified as a variant of concern (VOC) by the WHO on 26 November 2021 (Burki, 2022; Fan et al., 2022). Attenuated replication and pathogenicity were identified and potentially associated with the altered TMPRSS2-usage by this variant, impacting its infectivity (Goutam Mukherjee et al., 2022; Guo et al., 2022; McCallum et al., 2022; Meng et al., 2022; Shuai et al., 2022). When compared to the Delta variant, which preceded Omicron BA.1, the omicron variant presented higher affinity for ACE2, preferentially taking the endocytic pathway. This represents a concern about previously established cellular models becoming less efficient with the emergence of new variants and, in this way, the scientific community must remain vigilant.

It has also been shown that serial passage of a SARS-CoV-2 isolate in human cell lines can lead to a selection of clones that significantly improve infectivity in the human liver (Huh7 and Huh7.5) and lung cancer cells (Calu-1 and A549) (Ramirez et al., 2021). However, this selection does not take place without substantial mutations of the viral genome occurring, including spike protein mutations such as 9-amino-acid deletion and 3-amino acid changes (E484D, P812R, and Q954H) that exhibited significantly less dependence on ACE2 (Ramirez et al., 2021). Finding the balance between adaptations and the isolates closest to circulating variants is challenging for researchers.

## Other *in vitro* approaches

Cell lines are not the only *in vitro* tools for studying viruses. Although these platforms have certain strengths, especially as they can be stored indefinitely in the laboratory, are fast growing, and are usually well characterised, cell lines often differ genetically and phenotypically from their tissue of origin (Pan et al., 2009; Geraghty et al., 2014; Lorsch et al., 2014). Therefore, their results may be relatively distant from what is observed in the original tissues, and inferences must be cautiously made.

To come to more consistent conclusions, it is desirable that the researcher can combine different *in vitro* approaches in order to later complement them with *in vivo* approaches. Therefore, in addition to searching for cell lineage models for the study of SARS-CoV-2, we also sought to evaluate and develop alternative *in vitro* tools against this virus, such as assays in primary cells and cellular organoids.

### Primary cells

The primary cell culture is established from growing cells from the mechanical or enzymatic breakdown of tissue, from the moment of isolation until the first subculture (Lednicky and Wyatt, 2012; Alves and Guimarães, 2010). After disaggregation, the cells will be selected and maintained in culture, and they will be considered as cells which have been isolated from a given tissue. Although these cells have a short life span, their main advantage is the presence of their genotypic and phenotypic characteristics. That may reflect *in vitro* more faithfully than in the cell lines, as what happens in infected human airways.

For example, primary cell lines have been mainly used for SARS-CoV-2 culture in the aim to study the host transcriptional profiles, especially cytokines expression have been exhaustively analyzed aiming to better understand the immune phase and cytokine storm of COVID-19 (Blanco-Melo et al., 2020; Gamage et al., 2020). SARS-CoV-2 infection of primary neuronal cultures from transgenic mice expressing human ACE2 under the cytokeratin 18 promoter pointed for an activation of the ZBP1/pMLKL-regulated necroptosis pathway on this SARS-CoV-2 infected cells (Rothan et al., 2022). Proposing insights into the neuropathogenesis of SARS-CoV-2 infection in mice models.

Since the beginning of the SARS-CoV-2 outbreak, primary cells from the human airway epithelium (HAE) have been employed, since they had already been proposed for other coronaviruses. Primary HAE cells were used to isolate and discover SARS-CoV-2 just after its emergence in Wuhan, China. Bronchoalveolar lavage samples from three patients with unknown pneumonia were inoculated into the primary culture of HAE expanded in an air-liquid interface (ALI) system (Zhu et al., 2020). The virus was detected by transmission electron microscopy and molecular techniques for detecting the coronavirus genome. In addition, the culture presented CPE, the absence of ciliary movement, 96 hours post-infection (Zhu et al., 2020).

In Primary Human Nasal Epithelial Cells (HNECs) and human bronchial epithelial cells (HBECs), significant expression of ACE2 and TMPRSS2 was demonstrated (Gamage et al., 2020; Lukassen et al., 2020). The HNECs were permissive to SARS-CoV-2 strain harbouring a 382-nt deletion in ORF8, but revealed similar viral kinetics and host transcriptional profiles, as well as secretion of IP-10 (Gamage et al., 2020). Electron microscopy also sought to characterise the replication cycle, especially the assembly in viral factories of SARS-CoV-2 in HNEC (Morrison et al., 2022; Pinto et al., 2022). The infection of HBECs by SARS-CoV-2 led to the downregulation of tight junction molecules, the loss of cilia (Hao et al., 2020; Ryu and Shin, 2021), and the induction of many pro-inflammatory cytokines/chemokines (Blanco-Melo et al., 2020).

Primary human tracheal airway epithelial cells (HtAECs) and human small airway epithelial cells (HsAECs) also grow in ALI, showed themselves to be SARS-CoV-2 permissive cells, and presented robust viral release through the apical side for over 14 days post-infection (Nguyen et al., 2021). Both cells have been suggested to be useful for drug screening in antiviral trials (Nguyen et al., 2021).

The primary HAE cells are essential for understanding the pathophysiological mechanisms of SARS-CoV-2 infection and a large volume of information is generated by studies that use primary cells. However, it is challenging to reach general conclusions since the expression level of the evaluated molecules, which includes the receptors for SARS-CoV-2, vary by type, function, and location of the airway epithelial cells. They also differ from host to host depending on age, sex, and comorbid diseases (Ryu and Shin, 2021).

Although primary HAE cells have gained prominence in studies of SARS-CoV-2, which is a respiratory virus, other primary cultures, such as primary human renal epithelial cells (Kohli et al., 2022), primary ocular cells (Eriksen et al., 2021 - preprint; Eriksen et al., 2022), human peripheral blood mononuclear cells (PBMCs) (Pontelli et al., 2022) and human pancreatic progenitors (Szlachcic et al., 2022) are permissive to SARS-CoV-2. The range of primary cells permissive to SARS-CoV-2 opens the way for *in vitro* studies of more complex cellular organisations, such as organoids.

### Organoid culture

An organoid is a three-dimensional multicellular *in vitro* tissue construct grown from stem cells on an extracellular matrix-like scaffold with specific niche factors (Ranga et al., 2014; Clevers, 2016; Sridhar et al., 2020; Hautefort et al., 2022) They can self-renew and aim to mimics their corresponding *in vivo* organ, generating *in vitro* functional structures containing the cell types present in the tissue they model (Hautefort et al., 2022). Therefore, organoids represent a valuable tool for studying aspects of an organ in the tissue culture dish.

Organoid technology was quickly embraced as a powerful tool for human virus studies, as it promised to provide a more accurate picture of the host factors essential for establishing viral infection and the mechanisms of viral pathogenesis (Clevers, 2020; Sridhar et al., 2020). With the emergence of COVID-19, multiple research groups turned to organoid approaches, especially to understand the tissue tropism of SARS-CoV-2 (Tran et al., 2022).

As for the study of primary cells, many approaches aimed to establish a respiratory model for SARS-CoV-2. The multipotent adult tissue stem cells (ASCs) approach allowed the elaboration of human distal lung organoids with apical-out polarity to present ACE2 on the exposed external surface, producing SARS-CoV-2 infective particles that were tested in Vero E6 cells, recovering from the organoid supernatant titers of less than 100 PFU/mL (Salahudeen et al., 2020). Meanwhile, bronchial infection rarely shows positive cells for the SARS-CoV-2 Spike protein, according to immunohistochemical analysis (Sano et al., 2022).

When the infection is successful, the main advantage presented by ALI cultures of airway organoid systems is that the viral progeny recovered from them do not present culture-adaptive mutations in the multibasic cleavage site of the Spike protein of SARS-CoV-2 (Lamers et al., 2021; van der Vaart et al., 2021), suggesting that the organoid culture accurately models viral target cells *in vivo*.

As is the case for the cell lines derived from renal (HEK 293T), hepatic (Huh-7), and intestinal (Caco-2) tissues, kidney organoids established from human-induced pluripotent stem cells (iPSCs) (Monteil et al., 2020), ASCs-derived intestinal (Lamers et al., 2020; Zang et al., 2020; Zhou et al., 2020a) and liver organoids (Zhao et al., 2020a), were shown to be permissive to SARS-CoV-2. Infection of the liver organoids led to the formation of large syncytia such as CPE (Zhao et al., 2020a).

Established organoids from non-respiratory tissues play an essential role in understanding the viral pathophysiology and spread in the human body (Clevers, 2020). This can be further explored if organoids permissive to SARS-CoV-2 are developed from organs of which no cell lines permissive to the virus are known but which are affected in the infection process promoted by the emerging virus.

This is the case of eye cells. Cell lines have not been established which are derived from the tissues that make up the eyes, such as the cornea, sclera, and limbus. ACE2 and TMPRSS2 are expressed in the eyes, and in professional breeder cultures, SARS-CoV-2 has successfully infected limbus cells in particular (Eriksen et al., 2021; Tran et al., 2022). A similar result was obtained with retinal organoids and eye organoids, confirming endogenous receptors and SARS-CoV-2 infection mainly in limbus-like cells (Eriksen et al., 2021; Eriksen et al., 2022).

The issue of neurological complications of SARS-CoV-2 has also been addressed. It is proposed that the SARS-CoV-2 can enter the central nervous system by promoting damage to the epithelium and loss of barrier function or even by crossing the neural-mucosal interface in olfactory mucosa (Meinhardt et al., 2020). This may explain the loss of smell and taste experienced by some individuals with COVID-19.

The cell line U251 (derived from human glioblastoma) was previously suggested as a permissive cell for SARS-CoV-2 (Chu et al., 2020) but appears to have been little explored. In contrast, brain organoids have been well investigated, aiming to confirm or deny SARS-CoV-2 infection and identify target cell types in the brain. The results, however, have been conflicting, detecting the SARS-CoV-2 virus in the neurons but not producing infection (Ramani et al., 2020). Furthermore, effective infections were only confirmed in subsequent studies with brain organoids that contained choroid plexus epithelium and only in this choroid region (Jacob et al., 2020; Pellegrini et al., 2020).

Even though they are promising tools for studying SARS-CoV-2 or viruses in general, these cultures are still far from perfect (De Souza, 2018). Although more complex than the other *in vitro* models usually available in research laboratories, they are still not complex enough, typically lacking vasculature and immune cells, for example. This approach is not easy to set up, it can take some time to develop the technology, and the final material is sometimes quantitatively limited and hard to handle. Thus, it is unlikely to be used for routine screening and has only been developed to address some very precise questions. Some types of organoids also do not fully replicate the structure of the organ they model. Taking brain organoids as an example, anatomical markers are not possible as they occur in the *in vivo* brain. Finally, the cells in the hPSC-derived organoids are relatively immature, with expression profiles similar to foetal tissues (De Souza, 2018). These limitations, therefore, should be considered when choosing organoids to study SARS-CoV-2.

## Conclusions

**Figure 4:**
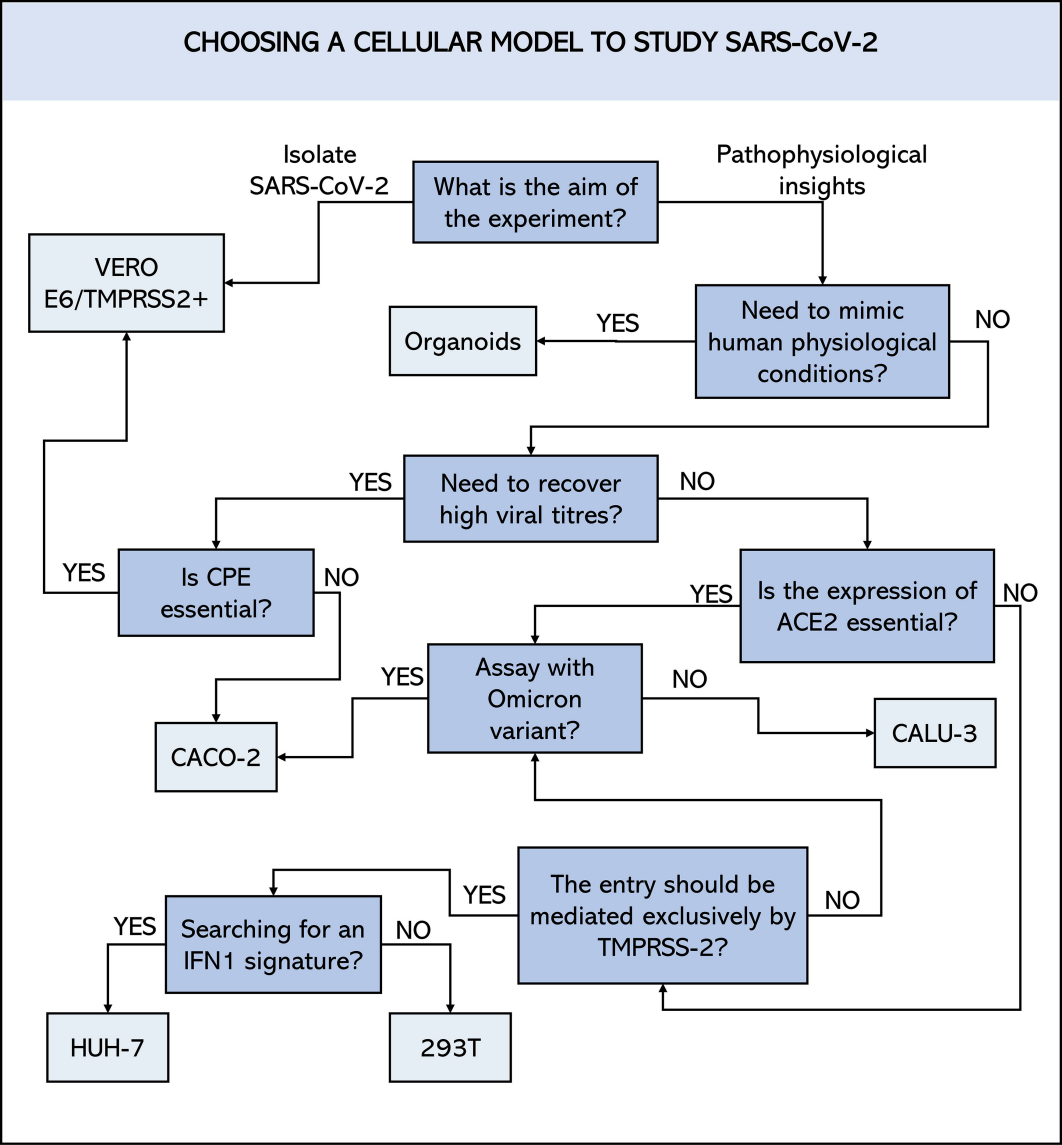
Schematic representation of a decision flowchart to choose which cell line to employ in SARS-CoV-2 studies. ACE2, angiotensin-converting enzyme 2 receptor; CPE, cytopathic effect; TMPRSS2, transmembrane protease serine 2; IFN1, Interferon type I.

There is currently no perfect cellular model for studying SARS-CoV-2. Each of the cells or approaches mentioned above has advantages over the others but also presents limitations in given setting. It is up to the researcher to find the balance between them and sometimes to consider overcoming these limitations through other resources. To this end, researchers must establish clear objectives and identify, within the current possibilities, the best model for the *in vitro* tests that will best serve their objective (**Figure 4**). This includes considering any financial and/or structural limitations. Given all the possibilities presented above, we hope that this study will contribute to making this choice easier.

## Author contributions

Conceptualisation: GAPS and BLS; writing— original draft preparation, GAPS, MLB, SA, CD and BLS; writing—review and editing: GAPS, CB, NW, SA, PC, CD and BLS; supervision: BLS; project administration, BLS. All authors have read and agreed to the published version of the manuscript.

## Funding

Agence Nationale de la Recherche (“Investments for the Future” program Méditerranée-Infection 10-IAHU-03).

## Acknowledgments

Special thanks to my laboratory colleagues, Julie Dergham, Rita Jaafar, and Wahiba Bader, for all their support and for helping me conceptualize the figures with their advice.

## Conflict of interest

The authors declare that the research was conducted in the absence of any commercial or financial relationships that could be construed as a potential conflict of interest.

## Publisher’s note

All claims expressed in this article are solely those of the authors and do not necessarily represent those of their affiliated organizations, or those of the publisher, the editors and the reviewers. Any product that may be evaluated in this article, or claim that may be made by its manufacturer, is not guaranteed or endorsed by the publisher.
