# Supplementary material for: Choosing a cellular model to study SARS-CoV-2
Source: Front Cell Infect Microbiol. 2022 Oct 21;12:1003608. doi: 10.3389/fcimb.2022.1003608 (PMC9634005; doi:10.3389/fcimb.2022.1003608)
Supplement: Supplementary file 1 [file Table_1.pdf]

SUPPLEMANTARY TABLE 1. Potential anti-SARS-CoV-2 restriction factors and their role in impairing the viral replication cycle

Antiviral Restriction Factor	Abbreviation	Step of SARS-CoV-2 cycle affected	Effect over SARS-CoV-2 replication	References
Lymphocyte Antigen 6 Family Member E	LY6E	Entry	Reduced entry of the pseudovirus expressing SARSCoV-2 S protein (VSV-S) compared to parental HEK 293T cells (>50%)	Martin-Sancho et al., 2021; Zhao at al., 2020 ; Pfaender et al., 2020.
Ubiquitin D	UBD			Martin-Sancho et al., 2021
Family With Sequence Similarity 46 Member C	FAM46C			
C-Type Lectin Domain Family 4 Member D	CLEC4D		Reduced entry of the pseudovirus expressing SARSCoV-2 S protein (VSV-S) compared to parental HEK 293T cells (>50%) and induced the expression of interferon induced protein with tetratricopeptide repeats 2 (IFIT2)	Martin-Sancho et al., 2021
E74 Like ETS Transcription Factor 1	ELF1			
REC8 Meiotic Recombination Protein	REC8			
p41 isoform of the invariant chain	CD74 p41		Stymie the endosomal entry of SARS-CoV-2 in Vero E6 cells	BRUCHEZ et al., 2020
Interferon Induced Protein with Tetratricopeptide Repeats 3	IFIT3	Translation and RNA replication	Reduced SARS-CoV-2 RNA levels (>50%) in HEK 293T cells	Martin-Sancho et al., 2021
Spermatogenesis Associated Serine Rich 2 Like	SPATS2L			
DnaJ Heat Shock Protein Family (Hsp40) Member C6	DNAJC6			
Regulator Of G Protein Signaling 2	RGSS2			
Uncharacterized/hypothetical gene	LOC152225			
Z-DNA Binding Protein 1	ZBP1			
Beta-1,4-Galactosyltransferase 5	B4GALT5			
Death domain-associated protein 6	DAXX		Basal expression of DAXX limited the SARS-CoV-2 replication, and DAXX over-expression further restricted infection. DAXX-mediated restriction of SARS-CoV-2 in A549-ACE2+ cells. This is antagonized by SARS-CoV-2 Papain-Like Protein.	Mac Kain et al., 2022

Zinc finger antiviral protein	ZAP		Overexpression of ZAP-L in HEK 293T, already overexpressing ACE2, reduced viral RNA yields in a dose-dependent manner. Gene depletion leads to increased SARS-CoV-2 RNA levels in Calu-3 cells.	Nchioua et al., 2020; Lee et al., 2021
Poly(ADP-Ribose) Polymerase Family Member 12	PARP12		Gene depletion leads to increased SARS-CoV-2 RNA levels in Calu-3 cells.	Lee et al., 2021
Tripartite Motif Containing 25	TRIM25			
CUGBP Elav-Like Family Member 1	CELF1			
Shiftless Antiviral Inhibitor Of Ribosomal Frameshifting	SHFL			
2'-5'-oligoadenilato sintetase 1	OAS1		Inducible CRISPR activation of OAS1 expression by resulted in a significant decrease in the production of SARS-CoV-2 progeny 2 in A549-ACE2+ cells.	Danziger et al., 2022
ER Lipid Raft Associated 1	ERLIN1	Assembly	Strongly attenuated late stages of SARS-CoV-2 replication in HEK 293T cells.	Martin-Sancho et al., 2021
Reticulophagy Regulator 1	RETREG1		Restricted SARS-CoV-2 replication in HEK 293T cells.	
Fibronectin Type III Domain Containing 4	FNDC4			
Bone Marrow Stromal Cell Antigen 2	BST2	Budding and release	Identified as an inhibitor of SARS-CoV-2 replication (>53%) and a potent inhibitor of viral release (>74%) in HEK 293T cells. This is antagonized by SARS-CoV-2 Orf7.	Martin-Sancho et al., 2021
