# Supplementary material for: Choosing a cellular model to study SARS-CoV-2
Source: Front Cell Infect Microbiol. 2022 Oct 21;12:1003608. doi: 10.3389/fcimb.2022.1003608 (PMC9634005; doi:10.3389/fcimb.2022.1003608)
Supplement: Supplementary file 2 [file Table_2.pdf]

SUPPLEMANTARY TABLE 2. SARS-CoV-2 non-permissive cells lines. Commonly used laboratory mammalian cell lines which do not support SARS-CoV-2 replication.

CELL LINE	ORGANISM	TISSUE	MORPHOLOGY	REFERENCES
A549	Human (*Homo sapiens*)	Lung	Epithelial	WANG et al., 2021
ECV304		Urinary bladder	Epithelial/endothelial	WURTZ et al, 2021
HCT-8		Colon	Epithelial	WURTZ et al, 2021
HEL		Lung	Fibroblast	WANG et al., 2021
HeLa		Cervix	Epithelial	WANG et al., 2021; WURTZ et al, 2021
HeLa229		Cervix	Epithelial	WANG et al., 2021
HEp2		Cervix	Epithelial	WANG et al., 2021; WURTZ et al, 2021.
HL-60		Peripherical blood	Lymphoblast	WURTZ et al, 2021
HT-29		Colon	Epithelial	WURTZ et al, 2021
MRC-5		Lung	Fibroblast	WANG et al., 2021; WURTZ et al, 2021
MRHF		Foreskin	Fibroblast	WANG et al., 2021
NCI-H292		Lung	Epithelial	WANG et al., 2021
THP1		Peripherical blood	Monocytes	WURTZ et al, 2021
RD		Muscle	Spindle; multinucleated	WANG et al., 2021
WI-38		Lung	Fibroblast	WANG et al., 2021
L929	Mouse (*Mus musculus*)	Subcutaneous areolar and adipose	Fibroblast	WURTZ et al, 2021
McCoy		Unknown	Fibroblast	WANG et al., 2021; WURTZ et al, 2021
MNA		Nerve	Neuroblastoma	WANG et al., 2021
P38D1		Lymphoma	Lymphoblast	WURTZ et al, 2021
RAW 264.7		Blood	Monocyte/Macrophage	WURTZ et al, 2021
DH82	Dog (*Canis familiaris*)	Malignant histiocytosis	Macrophage-like	WURTZ et al, 2021

MDCK		Kidney	Epithelial	WANG et al., 2021; WURTZ et al, 2021
OA3.T	Sheep (*Ovis aries*)	Testis	Epithelial	WURTZ et al, 2021
MDOK		Kidney	Epithelial	WURTZ et al, 2021
XTC-2	African clawed frog (Xenopus laevis)	Tadpole	Epithelial	WURTZ et al, 2021
BHK21	Golden/Syrian hamster (*Mesocricetus auratus*)	Kidney	Fibroblast	WURTZ et al, 2021
BA 886	Cattle (*Bos taurus*)	Aorta	Endothelial	WURTZ et al, 2021
R05T	Egyptian fruit bat (*Rousettus aegyptiacus*)	Head of fetus	Endothelia	WURTZ et al, 2021
R06E		Fetus	Endothelia	WURTZ et al, 2021
TB1 Lu	(*Tadarida brasiliensis*)	Lung	Endothelia	WURTZ et al, 2021
